# STK25-induced inhibition of aerobic glycolysis via GOLPH3-mTOR pathway suppresses cell proliferation in colorectal cancer

**DOI:** 10.1186/s13046-018-0808-1

**Published:** 2018-07-11

**Authors:** Fan Wu, Pin Gao, Wei Wu, Zaozao Wang, Jie Yang, Jiabo Di, Beihai Jiang, Xiangqian Su

**Affiliations:** 1 Key laboratory of Carcinogenesis and Translational Research (Ministry of Education), Department of Gastrointestinal Surgery IV, Peking University Cancer Hospital & Institute, 52 Fucheng Road, Haidian District, Beijing, 100142 China; 2 Inner Mongolia People’s Hospital, Hohhot, 010010 China

**Keywords:** STK25, GOLPH3, Colorectal cancer (CRC), Glycolysis, mTOR

## Abstract

**Background:**

Serine/threonine protein kinase 25 (STK25) is critical in regulating whole-body glucose and insulin homeostasis and the accumulation of ectopic lipids. The Warburg effect, also known as aerobic glycolysis, is an essential metabolic characteristic of cancer cells. However, the effects of STK25 on aerobic glycolysis of cancer cells remain unexplored. The aim of this study is to investigate the role of STK25 in colorectal cancer (CRC) and to elucidate the underlying mechanisms.

**Methods:**

The influences of STK25 on the cell proliferation were evaluated by MTT and colony formation assays. The roles of STK25 in aerobic glycolysis were determined by glucose uptake and lactate production assays. The interaction between STK25 and GOLPH3 was detected by co-immunoprecipitation, GST pull-down, and His-tag pull-down assays. Western blot was used to measure the expression of glycolytic genes, and the status of kinases in mTOR pathway. Moreover, a xenograft mouse model was used to investigate the effects of STK25 in vivo. The prognostic significance of STK25 was analyzed using public CRC datasets by a log-rank test.

**Results:**

STK25 suppressed proliferation, glycolysis and glycolytic gene expression in CRC cells. STK25 interacted with GOLPH3 and mediated glycolysis through GOLPH3-regulated mTOR signaling. Consistent with these observations, silencing of STK25 promoted tumor growth and glycolytic gene expression in an in vivo xenograft mouse model. Moreover, high levels of STK25 correlated with favorable prognosis in patients with CRC.

**Conclusions:**

Our results demonstrated that STK25 negatively regulates the proliferation and glycolysis via GOLPH3-dependent mTOR signaling. Accordingly, STK25 could be a potential therapeutic target for the treatment of CRC.

**Electronic supplementary material:**

The online version of this article (10.1186/s13046-018-0808-1) contains supplementary material, which is available to authorized users.

## Background

Colorectal cancer (CRC) is one of the most common malignancies and the second leading cause of cancer-related death in the world [1, 2]. Although notable progress has been made in the diagnosis and treatment of CRC, there are still challenges in determining the molecular mechanisms of tumor invasion, metastasis, and recurrence [3]. Thus, there is an urgent need for a greater understanding of the genetic and epigenetic alterations that lead to tumorigenesis in CRC, to improve the efficacy of disease treatments, including surgical techniques, adjuvant radiation, chemotherapy therapy, and follow-up strategies.

The Warburg effect, or aerobic glycolysis, is a critical feature of cancer cells, which have the tendency to employ glycolysis, even in the presence of sufficient oxygen [4, 5]. To generate energy, cancer cells convert their glucose into lactate, supplying a primary route for the carbon source that is required for macromolecular biosynthesis. This process enables cancer cells to meet the increasing energetic demands needed by rapid tumor growth [4, 5]. Thus, determining underlying molecular mechanism of aerobic glycolysis activation in cancer may shed light on the initiation and progression of cancer to identify potential drug targets.

Serine/threonine protein kinase 25 (STK25), also known as YSK1 and SOK1, is a member of the germinal center kinase III (GCK III) subfamily of the sterile 20 (STE20) kinase superfamily [6, 7]. STK25 plays pivotal roles in several biological processes, including the regulation of cell polarization and migration, promotion of cell death under extreme stresses, and modulation of Golgi morphology [8–13]. Importantly, previous studies have demonstrated that STK25 regulates whole-body glucose and insulin homeostasis, and the accumulation of ectopic lipids [7, 14–19]. Transgenic mice that overexpress STK25 experience lower glucose utilization and insulin sensitivity and elevated lipid deposition in the liver and pancreas, compared to wild-type littermates, when fed a high-fat diet [7]. Conversely, in STK25 knockout mice, Amrutkar et al. reported improved systemic glucose tolerance and insulin sensitivity, as well as reduced ectopic lipid storage in the liver and skeletal muscle [14]. Moreover, in type 2 diabetic patients, STK25 mRNA levels are significantly elevated in skeletal muscle, compared with healthy volunteers with normal glucose tolerance [19]. Thus, STK25 is a potential drug target for metabolic disease. However, the role of STK25 in aerobic glycolysis in cancer cells remains less well characterized.

The PI3K/AKT/mTOR pathway contributes to various cellular processes, including protein synthesis, cell growth, survival, autophagy, and metabolism [20–24]. This signaling cascade is upregulated in several human cancers and plays a critical role in modulating aerobic glycolytic metabolism [25–28]. mTOR is composed of two multiprotein complexes: mTOR complex 1 (mTORC1) and mTOR complex 2 (mTORC2) [29]. mTORC1 stimulates HIF1α and glycolytic enzymes, which induce glycolysis [25]. mTORC2 enhances the acetylation of FoxO1 and FoxO3, and the release of c-Myc from a suppressive miR-34c-dependent network, which results in the upregulation of glycolytic metabolism in cancer [26]. Due to the function of mTOR in glycolysis during tumorigenesis, whether STK25 is involved in mTOR signaling is needed to be elucidated.

In this study, we investigated the role of STK25 in glycolysis in CRC cells and the underlying molecular mechanisms. Our study demonstrates that STK25 inhibits aerobic glycolysis and malignant phenotypes in CRC. The interaction between STK25 and GOLPH3 contributes to the regulation of glycolysis through the mTOR signaling pathway. Moreover, high STK25 expression correlated with a favorable prognosis in patients with CRC. Taken together, these findings suggest that STK25 could serve as a potential target in the treatment of cancers that correlate with dysregulated glycolysis that is induced by activation of mTOR signaling.

## Methods

### Cell lines and cell culture

The RKO, LoVo, HCT116, and SW480 human CRC cell lines were purchased from American Type Culture Collection (ATCC, Manassas, VA). Cells were maintained in RPMI-1640 or DMEM medium (HyClone) that was supplemented with 10% FBS (PAA), penicillin 100 units/mL, and streptomycin 100 μg/mL in a humidified atmosphere with 5% CO_2_ at 37 °C.

### Quantitative real-time PCR (qRT-PCR)

Total RNA was isolated from CRC cells and tissues using Trizol (Invitrogen). Synthesis of first-strand cDNA was performed with a reverse transcription kit (Promega, Madison, WI, USA). For the qRT-PCR, we used the SYBR Green PCR Master Mix system (Toyobo Co. Ltd., Osaka, Japan) with the primers that are listed in Additional file 1: Table S1. Expression was quantified as the level of the indicated genes relative to an internal control, GAPDH, by ΔCt method [30].

### Western blot analysis

Western blot was carried out as described [31]. The primary antibodies were used at the following dilutions: STK25 (1:1000, Cat #25821–1-AP, Proteintech), GOLPH3 (1:1000, Cat #19112–1-AP, Proteintech), PDHK1 (1:1000, Cat #3820, Cell Signaling Technology), HK2 (1:1000, Cat #2867, Cell Signaling Technology), LDHA (1:1000, Cat #2012, Cell Signaling Technology), AKT (1:1000, Cat #9272, Cell Signaling Technology), p-AKT (1:1000, Cat #9271, Cell Signaling Technology), mTOR Substrates Antibody Sampler Kit (1:1000, Cat #9862, Cell Signaling Technology), p70S6K (1:1000, Cat #2708, Cell Signaling Technology), p-p70S6K (1:200, Cat #9234, Cell Signaling Technology), and GOLPH3 (1:1000, Cat #19112–1-AP, Proteintech). The protein levels were normalized to β-actin (1,3000, Cat #A1978, Sigma-Aldrich). The densities of the proteins were quantified with ImageJ software.

### Plasmids and siRNA transfections

The cDNA of full-length STK25 was cloned into pCMV-3Tag-1A. The ON-target plus SMARTpool STK25 small inhibitor RNA (siRNA) (human: #L-004873-00-0005) was purchased from Dharmacon. GOLPH3 siRNAs and negative control siRNA were synthesized as described (Shanghai Gene Pharma, China) [30]. CRC cells were transiently transfected with plasmids or siRNAs using Lipofectamine™ 2000 (Invitrogen) according to the manufacturer’s instructions.

### Cell proliferation and colony formation assay

Cells were seeded into a 96-well plate at 0.5 × 10^4^ cells/well with complete medium at 37 °C. To determine the numbers of live cells, MTT reagent was added to each well at 0, 24, 48, 72, and 96 h, respectively. The spectrometric absorbance at 570 nm was detected with a microplate reader (Bio-Rad, USA). To determine clonogenic ability, the cells were seeded into a 6-well plate at 250 cells/well and incubated for 14 days. Cell colonies were stained with 0.01% crystal violet and counted. These experiments were performed in triplicate.

### Lactate production and glucose uptake assays

Cells that were transfected with plasmids or siRNAs were cultured in complete medium for 48 h. The culture medium and original medium were collected to determine lactate and glucose levels using the EnzyChrom™ L-Lactate Assay Kit (BioAssay Systems) and the Amplex Red Glucose/Glucose Oxidase Assay Kit (Invitrogen), respectively.

### Co-immunoprecipitation (co-IP) assay

For Co-IP of Flag-STK25 and Myc-GOLPH3, 1 μg of Flag antibody (Cat#F1804, Sigma-Aldrich, USA) or c-Myc antibody (Cat#631206, Clontech, USA) was incubated with 10 μL of rProtein A sepharose beads (GE Healthcare) for 2 h at 4 °C. Then, 500 μg of total protein in cell lysis buffer was added to the mixture and incubated for 4 h at 4 °C. The immunoprecipitates were washed three times with lysis buffer, after which the immunoprecipitates and inputs were analyzed by western blot using Flag antibody (1:1000) and c-Myc antibody (1:500).

### GST pull-down and his-tag pull-down assays

Human STK25 was inserted into the pET-28a(+) vector. The pGEX-4 T-1-GOLPH3 plasmid has been described [31]. His-STK25, GST-GOLPH3, and GST were expressed and purified in accordance with the manufacturer’s instructions (Amersham). Then, 10 μg of His-STK25 was mixed with 10 μg of GST-GOLPH3 or GST and incubated with glutathione sepharose 4B beads (GE Healthcare) and Ni-NTA agarose (QIAGEN), respectively, for the GST pull-down and His-tag pull-down assays. The pellets were washed three times and boiled in SDS loading buffer. The bound proteins were loaded onto a 12% SDS-polyacrylamide gel and examined by western blot with anti-GST and anti-His tag (Beijing Zhongshan Golden Bridge Biotechnology Co Ltd., China).

### Xenograft model

The animal studies were approved by the Animal Ethics Committee of Peking University Cancer Hospital & Institute and were carried out in accordance with institutional guidelines. LoVo-shSTK25 and LoVo-shControl cells (~ 5 × 10^6^) were injected subcutaneously in 4-week-old female BALB/c-nude mice. Tumor growth was measured using calipers twice per week. Tumor volume was calculated by the following formula: 0.5 × L × W^2^ [32]. At 4 weeks after tumor inoculation, the tumor weight was measured.

### MicroPET/CT imaging

MicroPET/CT imaging of mice was performed as described [33, 34]. In brief, xenograft-bearing mice were administered 100–200 μCi [^18^F] FDG via tail vein injection. The mice were scanned in a Super Argus PET/CT scanner (Sedecal, Spain). Images were displayed by an MMWKS Super Argus.

### Analysis of gene expression datasets

The normalized mRNA expression data and CRC sample clinical information were downloaded from GEO (http://www.ncbi.nlm.nih.gov/geo/). Differences in STK25 expression between CRC tissues and adjacent noncancerous tissues were analyzed using paired-sample *t*-tests. The patients in each dataset were classified as STK25-high and STK25-low according to their mRNA levels of STK25. Survival curves were determined using the Kaplan-Meier method. The comparisons between groups were investigated by log-rank test. Overall survival was calculated as the time from the primary surgery to death or date of last follow-up. Recurrence-free survival was defined as the time from the primary surgery to recurrence or death from any cause. All statistical analyses were performed using SPSS 13.0 (SPSS Inc., Chicago, IL, USA). A two-sided *P* value of < 0.05 was considered to be statistically significant [31].

## Results

### STK25 inhibits CRC cell growth

**Fig. 1:**
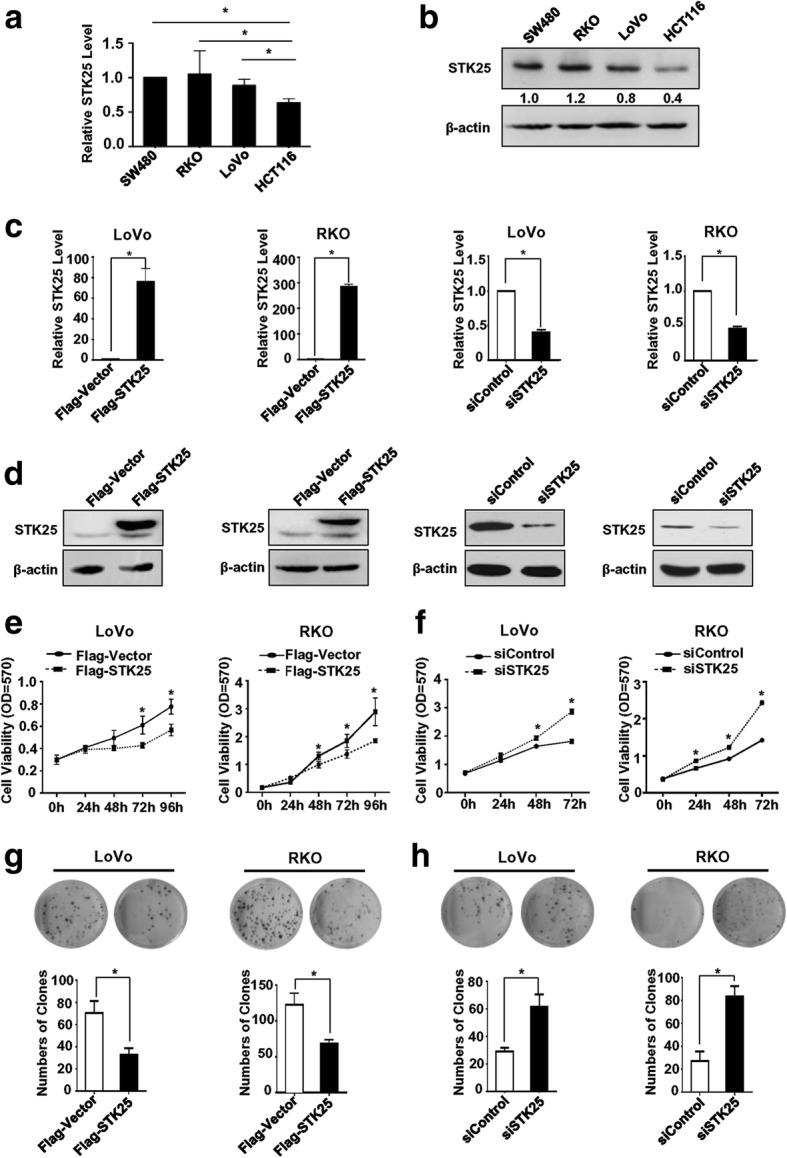
STK25 suppresses CRC cell proliferation. **a** Expression of STK25 mRNA in 4 CRC cell lines (SW480, RKO, LoVo, HCT116) was measured by qRT-PCR. **b** Expression of STK25 at the protein level in CRC cells was examined by western blot. Ratios of STK25:β-actin shown under the representative blots were normalized to STK25 in SW480 cells. Efficiency of STK25 overexpression and knockdown in LoVo and RKO cell lines were measured by qRT-PCR (**c**) and western blot (**d**). **e** Overexpression of STK25 in CRC cell lines significantly inhibits cell growth. **f** Depletion of STK25 significantly promotes cell proliferation. **g** STK25 overexpression attenuates colony formation in CRC cell lines. **h** Knockdown of STK25 increases colony formation in CRC cell lines. *, *P* < 0.05

The mRNA and protein levels of STK25 were determined in the CRC cell lines SW480, RKO, LoVo, and HCT116 using qRT-PCR and western blot, respectively. The expression of STK25 was higher in SW480 and RKO cells compared with LoVo and HCT116 cells at the mRNA and protein levels (Fig. 1a, b). Therefore, the RKO and LoVo lines, showing high and low expression of STK25, respectively, were used for further study. To investigate its function, STK25 was overexpressed and knocked down by transfecting CRC cells with STK25 plasmid and siRNAs, respectively. The efficiency of the overexpression and knockdown of STK25 was confirmed by qRT-PCR and western blot (Fig. 1c, d).

The effects of STK25 on CRC cell growth was measured by MTT and colony formation assays. The results suggested that overexpression of STK25 significantly decreases cell proliferation and colony formation in LoVo and RKO cells (Fig. 1e, g). However, knockdown of STK25 significantly promoted malignant phenotypes (Fig. 1f, h). These findings indicate that STK25 inhibits CRC cell growth.

### STK25 suppresses aerobic glycolysis and the expression of glycolytic genes in CRC cells

**Fig. 2:**
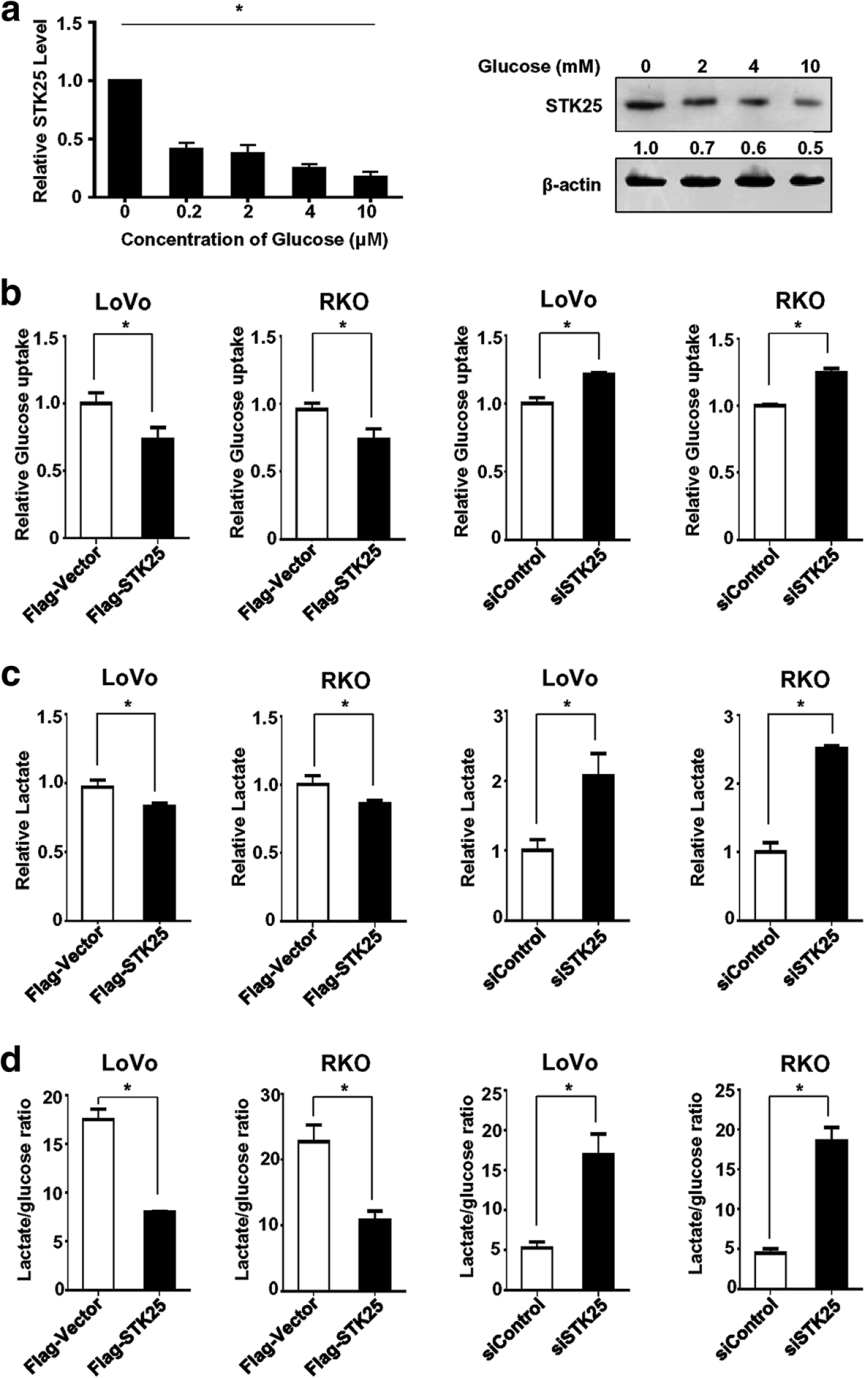
STK25 regulates aerobic glycolysis in CRC cells. **a** Changes in STK25 at the mRNA and protein levels were assessed by qRT-PCR (left panel) and western blot (right panel), respectively, under decreased glucose concentration. STK25:β-actin ratios shown under the representative blots were normalized to STK25 in LoVo cells deprived of glucose. Protein expression was normalized to β-actin. Culture media from controls and STK25-overexpressing or STK25-depleted CRC cells were analyzed to measure relative glucose uptake (**b**), lactate production (**c**), and lactate:glucose ratio (**d**). Data are expressed as mean ± SD. *, *P* < 0.05

To investigate whether metabolic stress influences STK25 expression in CRC cells, the changes in STK25 expression were measured under conditions of glucose starvation. The results showed that elevated expression of STK25 mRNA and protein was associated with reduced concentrations of glucose, as determined by qRT-PCR and western blot, respectively (Fig. 2a). The expression levels of STK25 increased after 48 h in response to glucose starvation, raising the possibility that STK25 regulates glucose metabolism.

To determine whether STK25 regulates aerobic glycolysis in CRC, glucose uptake and lactate production assays were performed. The analysis of extracellular metabolites showed that STK25 overexpression significantly decreased glucose consumption and lactate production, leading to a lower lactate: glucose ratio in CRC cells (Fig. 2b-d, left two panels). However, STK25 silencing markedly elevated glucose consumption, lactate production, and lactate: glucose ratios (Fig. 2b-d, right two panels). These findings suggest that STK25 suppresses aerobic glycolysis in CRC cells.

**Fig. 3:**
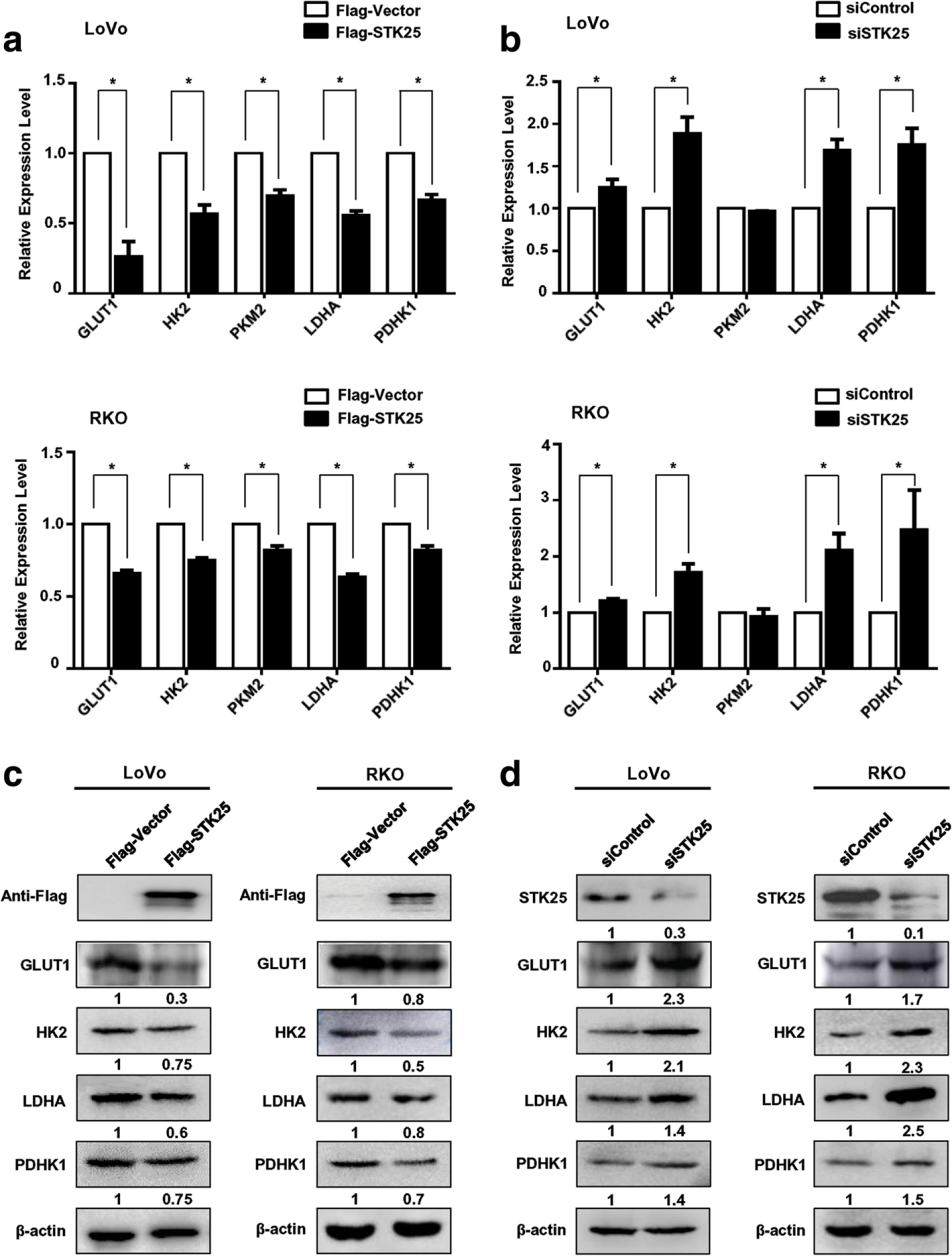
STK25 expression regulates glycolytic gene expression in CRC cells. The levels of glycolytic genes, including GLUT1, HK2, PKM2, LDHA, and PDHK1 were determined in controls and STK25-overexpressing or STK25-silenced CRC cells by qRT-PCR (**a**, **b**) and western blot (**c**, **d**). Data are expressed as mean ± SD. *, *P* < 0.05

To evaluate the effects of STK25 on the expression of glycolytic genes, qRT-PCR and western blot were performed to assess their levels, including GLUT1, HK2, PKM2, LDHA, and PDHK1. Overexpression of STK25 significantly decreased the expression of these glycolytic genes to varying degrees at the mRNA and protein levels in CRC cells (Fig. 3a, c), whereas knockdown of STK25 upregulated them (Fig. 3b, d). These data confirm the role of STK25 in regulating glycolytic metabolism.

### STK25 interacts with GOLPH3 and regulates its expression

STK25 localizes to the Golgi apparatus and is involved in the pathway that is required for cell migration and polarization [11, 12]. GOLPH3 is a peripheral membrane protein in the Golgi stack that mediates Golgi trafficking [35]. Thus, it has been postulated that there might be associations between STK25 and GOLPH3.

**Fig. 4:**
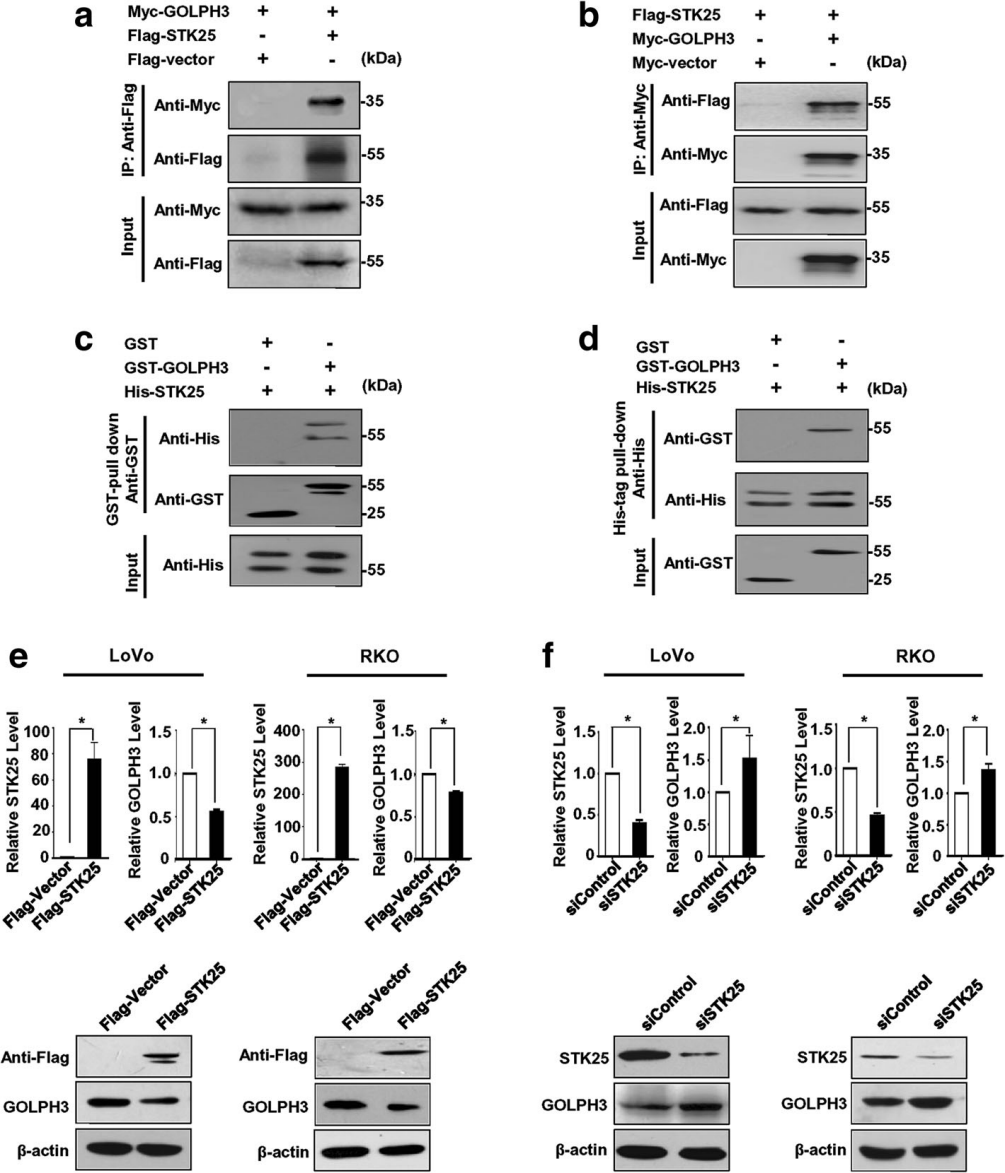
STK25 interacts with GOLPH3 and regulates its expression. **a**, **b** Exogenous STK25 interacts with GOLPH3. Cells were transfected with the indicated plasmids. Co-IP was performed using FLAG antibody to pull down FLAG-STK25 (**a**) or anti-Myc against Myc-GOLPH3 (**b**). Then, STK25 and GOLPH3 were detected with the indicated antibodies. **c**, **d** His-STK25 interacts directly with GST-GOLPH3 but not GST by in vitro GST pull-down and His-tag pull-down assays, respectively. **e** STK25 overexpression decreases GLOPH3 mRNA and protein levels in CRC cells. **f** STK25 knockdown increases GOLPH3 mRNA and protein levels in CRC cells

To examine the interaction between STK25 and GOLPH3, co-IP was performed, followed by western blot. Myc-GOLPH3 was detected in Flag-STK25 immune complexes (Fig. 4a). Similarly, Flag-STK25 was found in Myc-GOLPH3 immunoprecipitates by reciprocal IP (Fig. 4b). These findings suggest that STK25 can interact with GOLPH3 in vivo. Moreover, GST pull-down and His-tag pull-down assays were carried out to determine whether there is a direct interaction between STK25 and GOLPH3 in vitro. STK25 and GOLPH3 were bacterially expressed and purified as His-tag and GST fusion proteins, respectively. As shown in Fig. 4c, His-tagged STK25 was pulled down by GST-GOLPH3 but not by GST alone. Similarly, GST-GOLPH3, but not GST, bound to His-tagged STK25 (Fig. 4d), indicating direct binding between these proteins in vitro.

Next, to evaluate whether STK25 regulates GOLPH3 expression in CRC cells, we overexpressed or knocked down STK25 in CRC cells and measured GOLPH3 levels. STK25 overexpression significantly reduced the expression of GLOPH3 at the mRNA and protein levels (Fig. 4e), whereas STK25 knockdown upregulated it (Fig. 4f).

Moreover, to determine whether there is a feedback loop between STK25 and GOLPH3, we assessed whether GOLPH3 could regulate the expression of STK25. The results showed that overexpression or knockdown of GOLPH3 had little effects on STK25 protein levels in CRC cells (Additional file 2: Figure S1).

### STK25 mediates glycolysis via GOLPH3

**Fig. 5:**
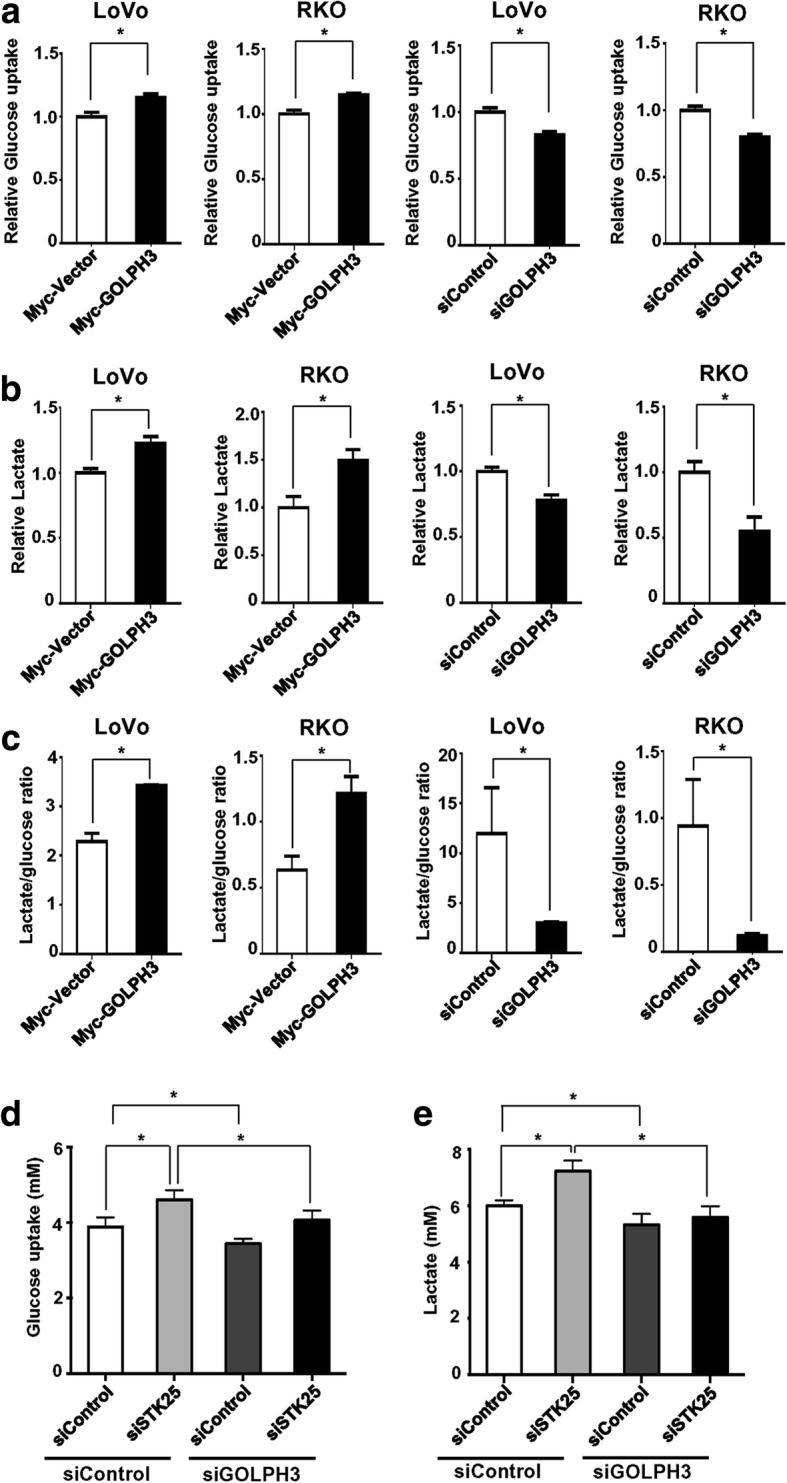
STK25 modulates glycolysis via GOLPH3. Culture media from controls and GOLPH3-overexpressing or GOLPH3-depleted CRC cells were analyzed to measure relative glucose uptake (**a**), lactate production (**b**), and lactate:glucose ratio (**c**). Knockdown of GOLPH3 decreases the promotion of glucose uptake (**d**) and lactate production (**e**) induced by STK25 depletion in LoVo cells. Data are expressed as mean ± SD. *, *P* < 0.05

STK25 has been shown to inhibit glycolysis in CRC cells. According to the association between STK25 and GOLPH3, we evaluated the role of GOLPH3 in glycolysis. GOLPH3 overexpression significantly promoted glucose consumption and lactate production, resulting in a higher lactate: glucose ratio in LoVo and RKO cells (Fig. 5a-c, left two panels). In contrast, GOLPH3 depletion impaired glucose consumption and lactate production, and lowered the lactate: glucose ratio (Fig. 5a-c, right two panels). These data indicate that GOLPH3 enhances aerobic glycolysis in CRC cells.

To further investigate whether STK25 regulates aerobic glycolysis through GOLPH3 in CRC cells, we knocked down STK25 and GOLPH3 using siRNAs in LoVo cells. As expected, STK25 depletion promoted glucose consumption and lactate production, which were impeded by knockdown of GOLPH3 in LoVo (Fig. 5d, e) and RKO cells (Additional file 3: Figure S2). These findings suggest that STK25 regulates aerobic glycolysis in part by modulating the expression of GOLPH3.

### STK25 modulates mTOR signaling through GOLPH3

**Fig. 6:**
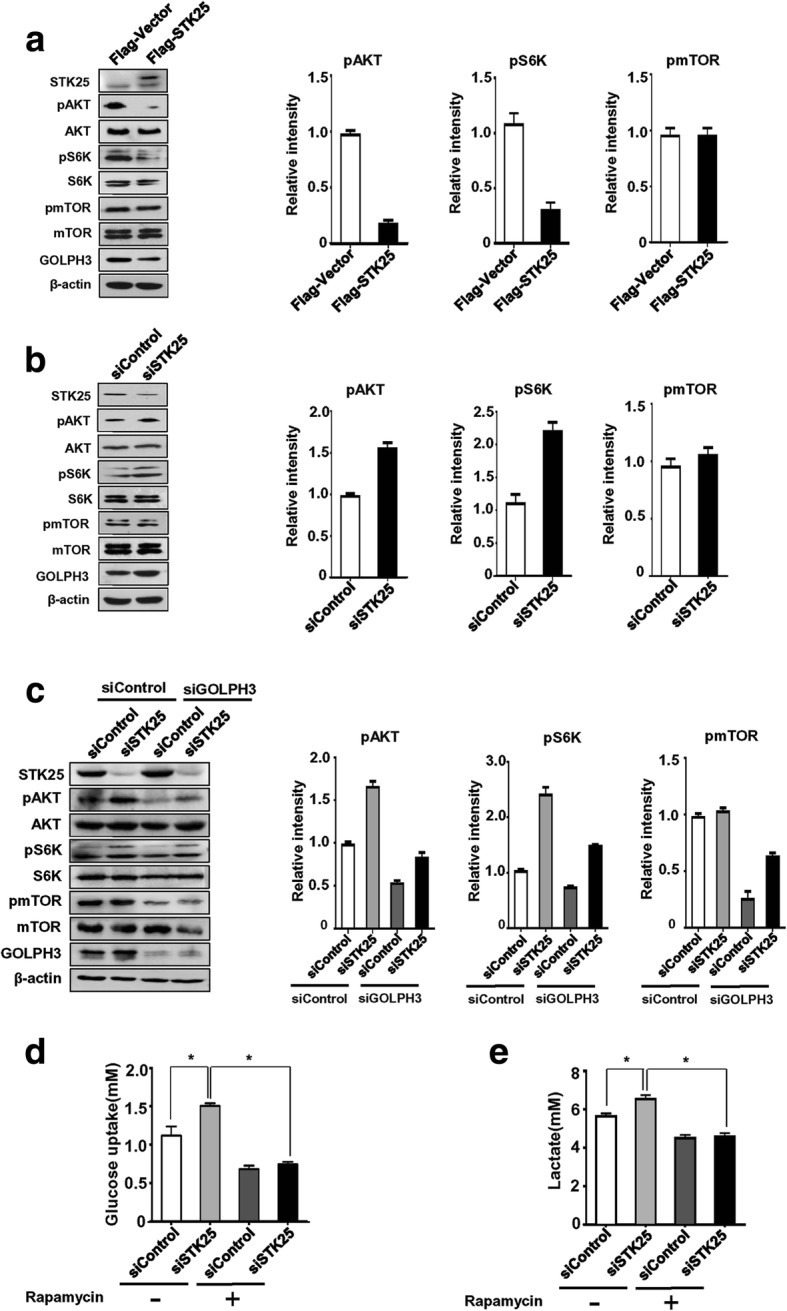
STK25 mediates glycolysis through GOLPH3-regulated mTOR signaling. Western blot was performed to assess the levels and phosphorylation of mTOR substrates in LoVo cells transfected with STK25 expression plasmid (**a**) or STK25 siRNA (**b**). **c** Knockdown of GOLPH3 partially reverses the upregulation of pAKT and pS6K induced by STK25 depletion. The relative phosphorylation levels of specific substrates of mTORC1 and mTORC2 were quantified by assessing the relative intensity of phosphorylated bands to corresponding protein bands (**a**-**c**; shown as mean ± SD of three scans). Rapamycin attenuates STK25 knockdown-induced glucose uptake (**d**) and lactate production (**e**). Data are expressed as mean ± SD. *, *P* < 0.05

GOLPH3 promotes growth factor-induced mTOR signaling in human cancer cells [35]. Due to the association between STK25 and GOLPH3, we sought to investigate whether STK25 regulates mTOR signaling in CRC cells. Since mTOR functions as two complexes mTORC1 and mTORC2, we examined the expression and phosphorylation status of direct substrate of mTORC1 and mTORC2, including AKT, S6K, and mTOR. STK25 overexpression suppressed AKT and S6K phosphorylation and lowered GOLPH3 levels in LoVo cells (Fig. 6a). However, STK25 knockdown enhanced the phosphorylation of AKT and S6K (Fig. 6b). No significant alterations in mTOR or pmTOR were observed (Fig. 6a, b).

Next, to determine the role of GOLPH3 in STK25-regulated mTOR signaling, we knocked down GOLPH3 and STK25 with siRNAs and measured the phosphorylation of AKT and S6K. GOLPH3 depletion suppressed the induction of AKT and S6K phosphorylation on STK25 silencing (Fig. 6c). These data raise the possibility that STK25 modulates mTOR signaling through GOLPH3.

### STK25 regulates glycolysis through mTOR signaling

mTOR signaling contributes to the activation of cellular glycolysis [25–27], and our data suggest that STK25 regulates glycolysis in part through mTOR signaling. To test this hypothesis, we abolished the activity of mTOR with rapamycin and knockdown of STK25 and measured glucose consumption and lactate production in CRC cells. As expected, rapamycin impaired glucose consumption and lactate production in a dose-dependent manner (Additional file 4: Figure S3). More importantly, rapamycin completely abrogated the promoting effects of STK25 depletion on glucose consumption and lactate production (Fig. 6d, e). Taken together, these results indicate that STK25 regulates glycolytic metabolism in CRC cells through the mTOR pathway.

### STK25 depletion promotes tumor growth and glycolytic gene expression in a xenograft mouse model

**Fig. 7:**
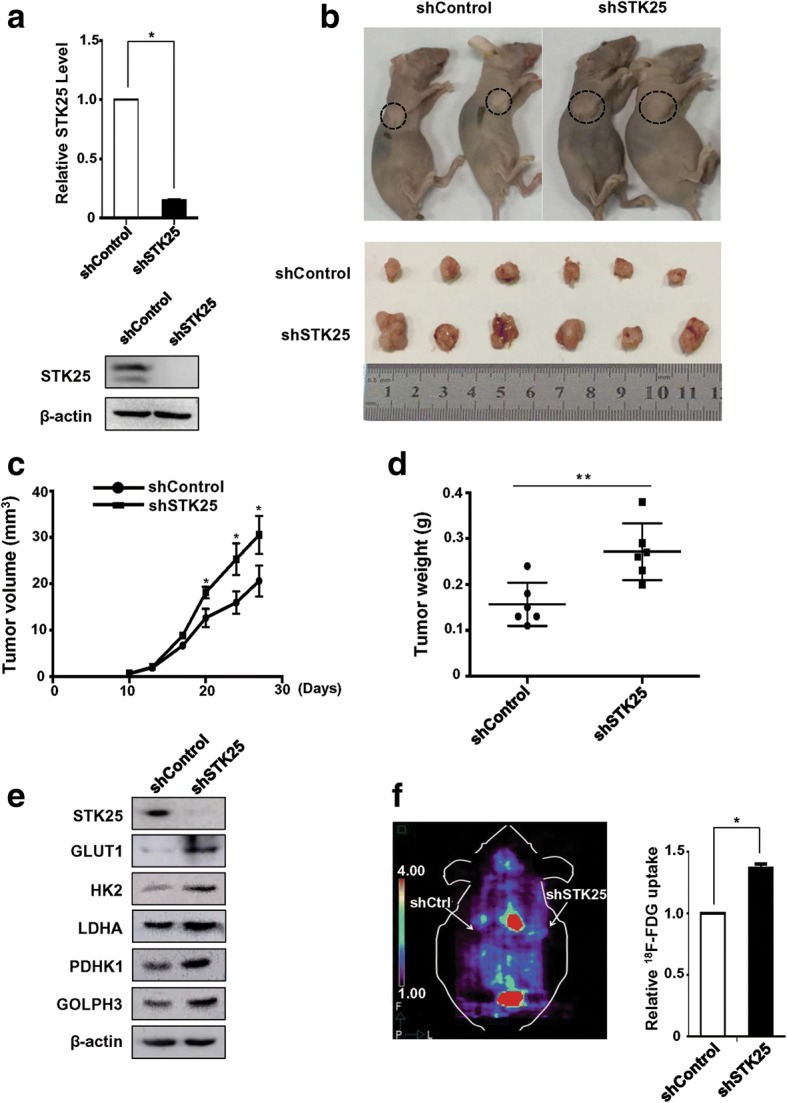
STK25 knockdown promotes tumor growth and glycolytic gene expression in a xenograft mouse model. **a** The stable knockdown of STK25 by a shRNA construct in LoVo cells was confirmed by qRT-PCR and western blot. **b** Representative images of tumor-bearing mice implanted with LoVo-shSTK25 or LoVo-shControl cells (upper panel). Tumor masses were harvested from the corresponding xenografts on Day 28 (lower panel). Tumor volumes (**c**) and tumor weights (**d**) were measured on the indicated days. **e** Lysates from tumor masses harvested from the indicated mice were analyzed by western blot for the expression of glycolytic genes. **f** MicroPET/CT imaging of mice. Representative PET/CT photographs of animals (left panel). Knockdown of STK25 promotes ^18^F-FDG uptake in mice bearing LoVo xenograft (right panel). Data are expressed as mean ± SD. *, *P* < 0.05. **, *P* < 0.01

To further investigate the correlation between STK25-mediated regulation of aerobic glycolysis and tumorigenesis, human CRC xenografts in nude mice were established by subcutaneous injection of LoVo-shSTK25 or LoVo-shControl cells. The transduction efficiency was assessed by qRT-PCR and western blot (Fig. 7a). Tumor growth and weight in the LoVo-shSTK25-injected group increased significantly compared with the LoVo-shControl-injected group (Fig. 7b-d).

Additionally, the tumor tissues were analyzed to evaluate the differences in STK25 and glycolytic gene expression. By western blot, lower STK25 expression in STK25 shRNA-expressing tumors was associated with upregulation of GLUT1, HK2, LDHA, and PDHK1 compared with shControl-expressing tumors (Fig. 7e), which is consistent with the in vitro data. To confirm the influence of STK25 on glycolysis, microPET/CT imaging of mice was performed to compare the uptake of ^18^F-FDG by the tumors. ^18^F-FDG accumulation was significantly enhanced by STK25 depletion (Fig. 7f). These findings indicate that the silencing of STK25 promotes tumor growth and increases glycolysis in tumors.

### High levels of STK25 suggest a better prognosis in patients with CRC

**Fig. 8:**
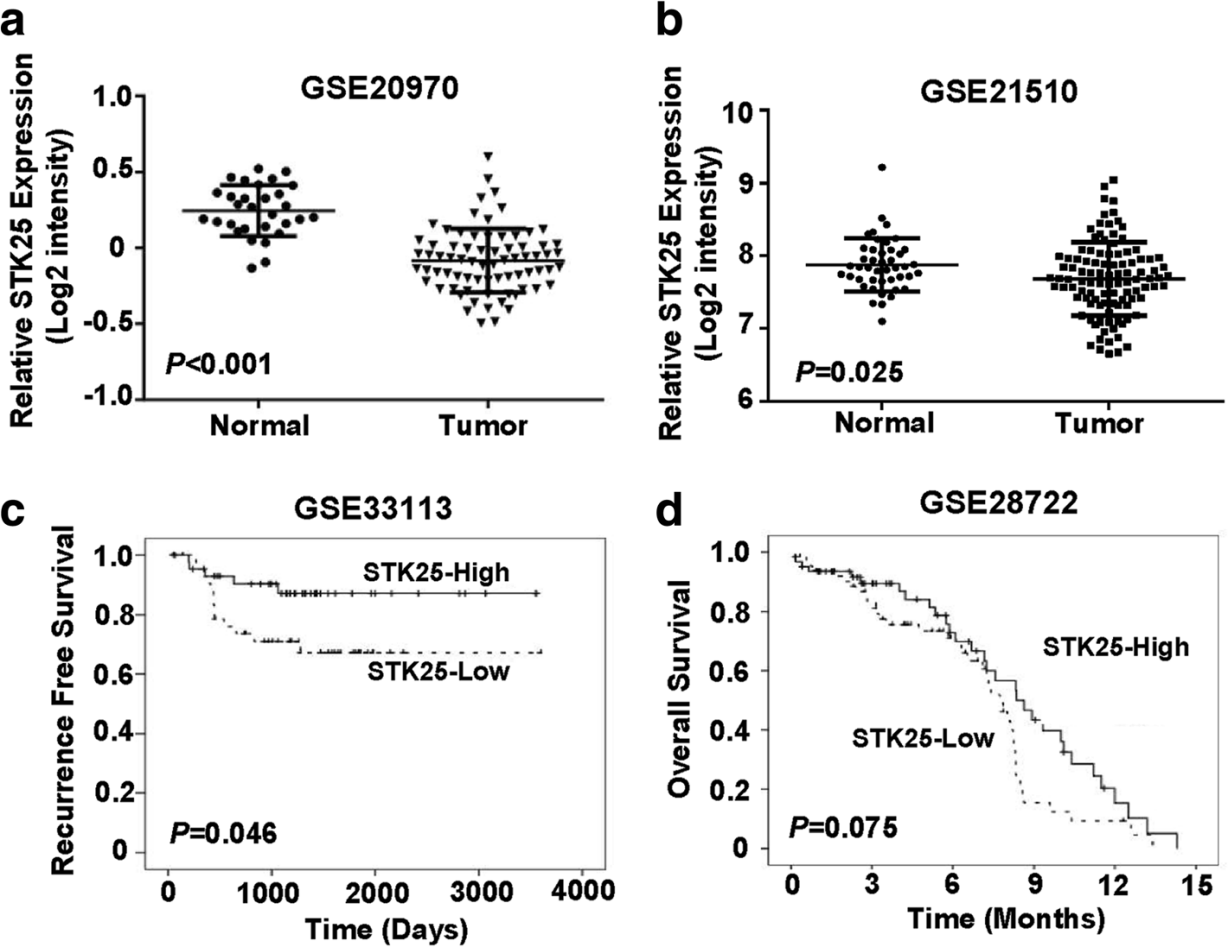
STK25 Expression in human CRC tissues. **a**, **b** STK25 mRNA levels are significantly lower in tumors than in adjacent noncancerous mucosal samples from the GEO datasets. Kaplan-Meier survival curves show that high levels of STK25 are associated with favorable survival in the GSE33113 (**c**) and GSE28722 (**d**) GEO datasets. *P* values were calculated using the log-rank test

Public datasets were downloaded to analyze STK25 expression in CRC tissues. In the GSE20970 and GSE21510 datasets, STK25 mRNA levels were significantly lower in tumors than in adjacent noncancerous tissues (*P* < 0.001 and *P* = 0.025, respectively, Fig. 8a, b).

According to STK25 mRNA levels, patients in each dataset were classified into STK25 high- and low-expressing groups, based on the median gene expression value. To determine the prognostic value of STK25 in survival, Kaplan-Meier survival plots were drawn and log-rank test were performed. In the GSE33113 CRC dataset, patients with STK25-high tumors had significantly longer recurrence-free survival than those with low levels (*P* = 0.046, Fig. 8c). Similarly, in the GSE28722 dataset, patients with elevated STK25 levels showed a borderline significant association with improved overall survival, compared with those with reduced levels (*P* = 0.075, Fig. 8d). These findings suggest that high levels of STK25 predict an improved prognosis for patients with CRC.

## Discussion

In the current study, we identified STK25 as a suppressor of proliferation and glycolysis in CRC cells. STK25 mediated glycolysis through its interaction with GOLPH3, modulating mTOR signaling. Moreover, high levels of STK25 indicated a favorable prognosis in patients with CRC. These findings suggest that STK25 inhibits aerobic glycolysis, thus impairing the proliferation of CRC cells.

As a critical signaling molecule, STK25 is involved in Golgi organization, cell migration and polarity, cell death and apoptosis, cardiovascular development, and energy metabolism [7–19, 36]. STK25 localizes to the Golgi apparatus and is activated via interaction with Golgi matrix protein 130 (GM130), contributing to cell migration and polarity [10, 11]. STK25 translocates from the Golgi apparatus to the nucleus in response to chemical anoxia and results in the regulation of cell death [12]. STK25 interacts with CCM3 (PDCD10), promotes apoptosis under oxidative stress, and controls endothelial cell junctions by regulating Rho via directly activating moesin, which contributed to cardiovascular development and human vascular disease [36]. Furthermore, STK25 is a new regulator of whole-body energy homeostasis, which is critical in glucose utilization, insulin sensitivity, and ectopic lipid deposition, through the control of triacylglycerol (TAG) synthesis in vivo and in vitro [7, 14–19]. Consequently, STK25 is a potential target for the treatment of type 2 diabetes [14].

According to the role of STK25 in regulating glucose utilization in human hepatocytes, we investigated whether STK25 has effects on glycolysis in CRC cells. Consistent with a previous study in human hepatocytes, which reported suppression of glucose uptake by overexpression of STK25 [16], our results suggest that STK25 attenuates aerobic glycolysis and downregulates glycolytic genes in CRC cells.

Accumulating evidence has demonstrated that mTOR pathway is crucial in aerobic glycolysis and tumorigenesis [20–28]. mTOR consists of two multi-protein complexes: mTORC1 and mTORC2 [29]. mTORC1 is a critical activator of glycolysis, upregulating PKM2, Glut3, and other glycolytic enzymes under normoxic conditions [27, 28]. mTORC2 has been reported to control glycolytic metabolism via FoxO acetylation and c-Myc upregulation in glioblastoma [26].

In the present study, we identified STK25 as a regulator and interactor of GOLPH3. GOLPH3 is identified as an oncoprotein, which is frequently amplified in several tumors [35, 37], and shuttles between Golgi apparatus and mitochondria to promote mitochondrial biogenesis and function in breast cancer cells, enhancing anabolic tumor growth [35, 38, 39]. Since GOLPH3 activates mTOR signaling through the phosphorylation of specific substrates of mTORC1 and mTORC2, increasing the sensitivity of tumor cells to rapamycin in vivo [35]. Thus, we investigated the effects of STK25 in regulating mTOR signaling. Our results suggested that STK25 modulates the mTOR cascade in a GOLPH3-dependent manner. Moreover, mTOR signaling is required for STK25-regulated glycolysis. Collectively, these results raise the possibility that STK25 promotes glycolysis by activating the GOLPH3-mTOR pathway.

The functions of STK25 in cancer development and progression are debated. A previous study suggested that STK25 is highly expressed in prostate cancer, compared with benign prostatic hyperplasia, contributing to prostate tumorigenesis [40]. However, in neuroblastoma patients, Costa et al. demonstrated that high STK25 levels in tumors are associated with a better prognosis and that STK25 acts as an effector of the death signaling pathway that is initiated by TrkA and CCM2 [41]. Consistent with the study by Coata et al., our analysis of public datasets indicates that high levels of STK25 correlate with a favorable clinical outcome in patients with CRC.

There are several limitations of this study. Although we demonstrated a correlation between STK25 and GOLPH3, which regulates mTOR signaling-dependent glycolysis, the effects of STK25 and GOLPH3 on rapamycin-mediated glycolysis should be investigated. Furthermore, to validate the clinical value of STK25 in patients with CRC, replication cohorts that include clinicopathological variables are required.

## Conclusions

In conclusion, our findings demonstrate that STK25 inhibits cell proliferation and glycolysis via GOLPH3-dependent mTOR signaling. Additionally, CRC patients with STK25-high tumors have improved survival. These data implicate STK25 as a potential target for the Warburg effect in cancer therapy.

## Additional files

Additional file 1: **Table S1.** Sequences of primers. (DOCX 18 kb)

Additional file 2: **Figure S1.** Overexpression or knockdown of GOLPH3 had little effects on STK25 protein levels. **a** GOLPH3 overexpression had little effects on STK25 protein levels in CRC cells. **b** knockdown of GOLPH3 had slightly effects on STK25 protein levels in CRC cells. (TIF 1182 kb)

Additional file 3: **Figure S2**. STK25 regulates aerobic glycolysis in part by modulating the expression of GOLPH3. Knockdown of GOLPH3 decreases the promotion of glucose uptake (**a**) and lactate production (**b**) induced by STK25 depletion in RKO cells. Data are expressed as mean ± SD. *, *P* < 0.05. (TIF 1010 kb)

Additional file 4: **Figure S3**. Rapamycin impaired glycolysis. Rapamycin inhibited glucose consumption (**a**) and lactate production (**b**) in LoVo cells. (TIF 881 kb)

## Abbreviations

ATCC: American Type Culture Collection
co-IP: Co-immunoprecipitation
CRC: colorectal cancer
FBS: Fetal bovine serum
GCK III: Germinal center kinase III
GM130: Golgi matrix protein 130
mTOR: Mammalian target of rapamycin
mTORC1: mTOR complex 1
mTORC2: mTOR complex 2
qRT-PCR: quantitative real-time PCR
STE20: sterile 20
STK25: Serine/threonine protein kinase 25
TAG: triacylglycerol.
