# Supplementary material for: STK25-induced inhibition of aerobic glycolysis via GOLPH3-mTOR pathway suppresses cell proliferation in colorectal cancer
Source: J Exp Clin Cancer Res. 2018 Jul 11;37:144. doi: 10.1186/s13046-018-0808-1 (PMC6042396; doi:10.1186/s13046-018-0808-1)
Supplement: Supplementary file 1 — Table S1. Sequences of primers. (DOCX 18 kb) [file 13046_2018_808_MOESM1_ESM.docx]

**Table S1. Sequences of primers.**

| **Primers** | **Sequences** |
| --- | --- |
| **STK25** |  |
| Forward primer | 5’-GCTCCTACCTAAAGAGCACCA -3’ |
| Reverse primer | 5’-TGGCAATGTATGTCTCCTCCAG -3’ |
| **GOLPH3** |  |
| Forward primer | 5’- AGGAAGCCGTTCTTGACAAATG -3’ |
| Reverse primer | 5’-GGCATGAGCCAGGTAAATGAG -3’ |
| **GLUT1** |  |
| Forward primer | 5’-CAGAAGGTGATCGAGGAGTTC -3’ |
| Reverse primer | 5’-AGAGAAGGAGCCAATCATGCC -3’ |
| **HK2** |  |
| Forward primer | 5’-ACAGAACACGGAGAGTTCCT -3’ |
| Reverse primer | 5’-GATGTCCTCAGGGATGGCATAG -3’ |
| **PKM2** |  |
| Forward primer | 5’-GAGATCCGAACTGGGCTCAT -3’ |
| Reverse primer | 5’-CACTTCCACCACCTTGCAGA -3’ |
| **LDHA** |  |
| Forward primer | 5’-GAGAGTGCTTATGAGGTGATC-3’ |
| Reverse primer | 5’-CCGTAAAGACCCTTAATATGG-3’ |
| **PDHK1** |  |
| Forward primer | 5’-GGACTTCGGATCAGTGAATG -3’ |
| Reverse primer | 5’-CCAATTGAACGGATGGTGTC -3’ |
| **GAPDH** |  |
| Forward primer | 5’-GGACTCATGACCACAGTCCATG -3’ |
| Reverse primer | 5’-CAGGGATGATGTTCTGGAGAGC -3’ |
