# Supplementary figures and images for: STK25-induced inhibition of aerobic glycolysis via GOLPH3-mTOR pathway suppresses cell proliferation in colorectal cancer
Source: J Exp Clin Cancer Res. 2018 Jul 11;37:144. doi: 10.1186/s13046-018-0808-1 (PMC6042396; doi:10.1186/s13046-018-0808-1)

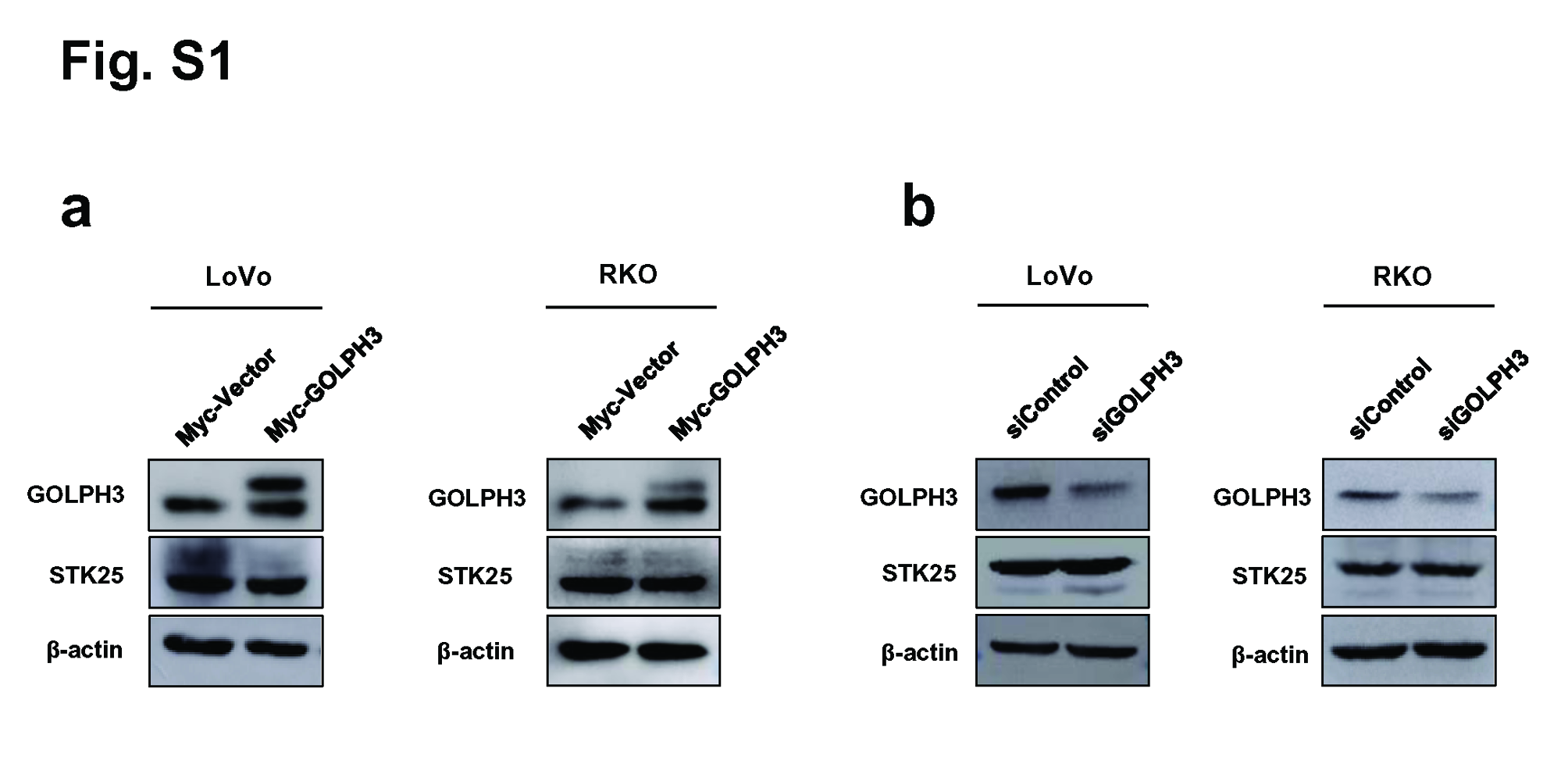

Supplement: Supplementary file 2 — Figure S1. Overexpression or knockdown of GOLPH3 had little effects on STK25 protein levels. a GOLPH3 overexpression had little effects on STK25 protein levels in CRC cells. b knockdown of GOLPH3 had slightly effects on STK25 protein levels in CRC cells. (TIF 1182 kb) [file 13046_2018_808_MOESM2_ESM.tif]

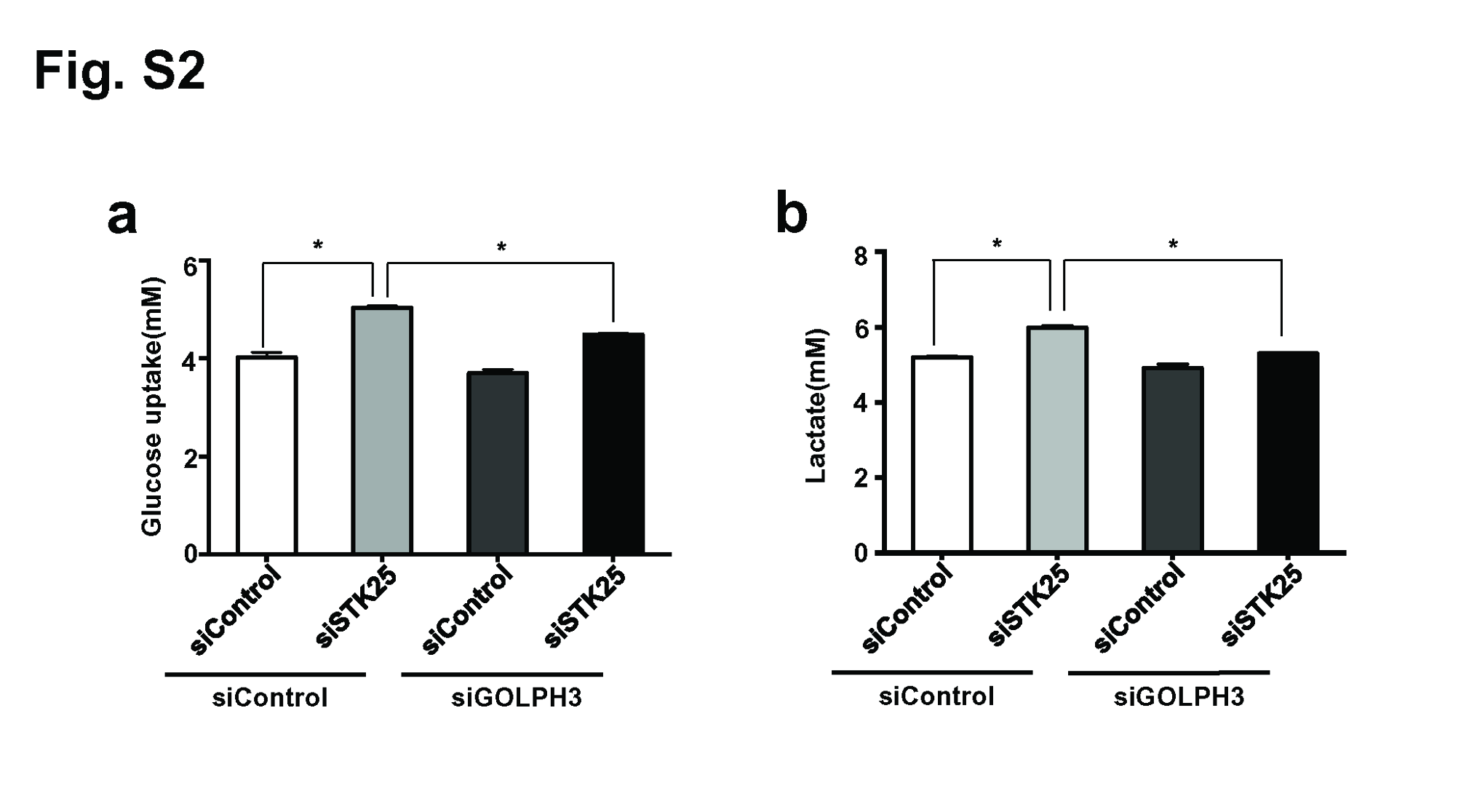

Supplement: Supplementary file 3 — Figure S2. STK25 regulates aerobic glycolysis in part by modulating the expression of GOLPH3. Knockdown of GOLPH3 decreases the promotion of glucose uptake (a) and lactate production (b) induced by STK25 depletion in RKO cells. Data are expressed as mean ± SD. *, P < 0.05. (TIF 1010 kb) [file 13046_2018_808_MOESM3_ESM.tif]

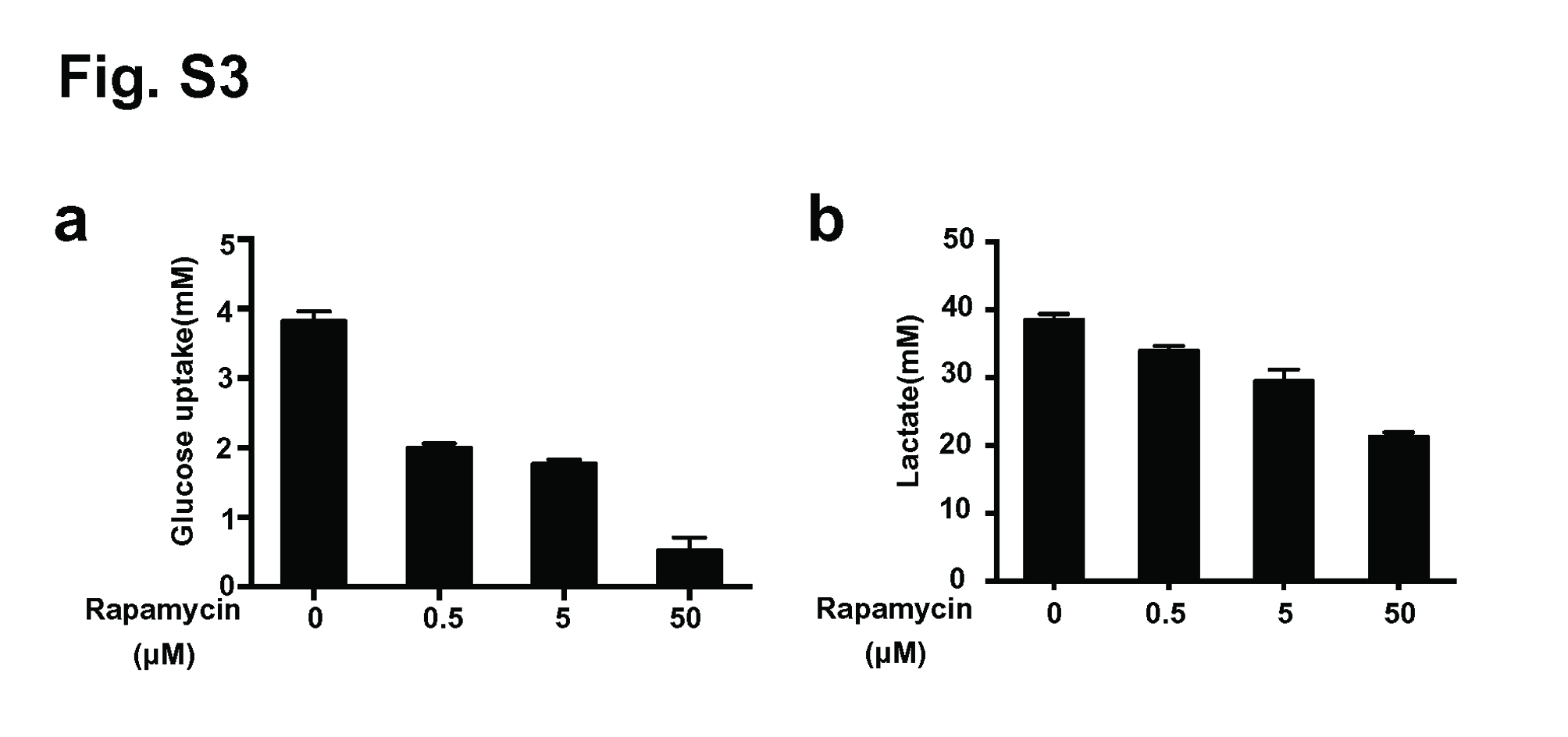

Supplement: Supplementary file 4 — Figure S3. Rapamycin impaired glycolysis. Rapamycin inhibited glucose consumption (a) and lactate production (b) in LoVo cells. (TIF 881 kb) [file 13046_2018_808_MOESM4_ESM.tif]
